# Comparison of the effect of ketorolac versus triamcinolone acetonide injections for the treatment of de Quervain’s tenosynovitis: a double-blind randomized controlled trial

**DOI:** 10.1186/s12891-022-05784-x

**Published:** 2022-09-01

**Authors:** Sitthiphong Suwannaphisit, Porames Suwanno, Warangkana Fongsri, Chaiwat Chuaychoosakoon

**Affiliations:** 1 Department of Orthopaedics, Faculty of Medicine, Vajira Hospital, Navamindradhiraj University, 681 Samsen Road, Dusit, 10300 Bangkok Thailand; 2 Department of Orthopedics, Faculty of Medicine, Prince of Songkla University, 15 Karnjanavanich Road, Songkhla 90110 Hat Yai, Thailand

**Keywords:** De Quervain’s tenosynovitis, Steroid injection, Non-steroid injection, Pain, Functional outcome

## Abstract

**Background:**

De Quervain’s disease is tenosynovitis of the first dorsal compartment causing severely painful radial-side wrist pain and impaired function. Steroids are effective in treating this condition due to their anti-inflammatory properties. However, this drug causes problems such as hypopigmentation, and is contradicted in diabetes mellitus patients. Non-steroidal anti-inflammatory drug (NSAID) which are efficacious in shoulder pathology and not contraindicated in diabetics and can be used to avoid the local effects of steroids could be beneficial for some patients. The present study was a randomized controlled trial to examine the differences in pain scores and functional response to local injections of a corticosteroid and the NSAID ketorolac.

**Methods:**

Sixty-four patients with radial styloid tenosynovitis were randomized using a computer-generated random number table into two groups receiving either a ketorolac injection or a triamcinolone injection. We evaluated post-injection pain intensity using a verbal numerical rating scale (VNRS), functional outcomes using the Thai Disabilities of the Arm, Shoulder and Hand (DASH) scale, and evaluated grip and pinch strengths, recorded at baseline and 6 weeks after the injection.

**Results:**

Thirty-one participants in the ketorolac group and 29 participants in the triamcinolone group completed the study and were included in the analysis. There were no significant differences in the assessments at baseline. At the 6-week conclusion of the study, patients in the triamcinolone group had a statistically lower average pain score than in the ketorolac group (0.7 ± 2.0 vs 5.3 ± 3.2, *P* < 0.001), higher DASH functional score (4.4 ± 6.5 vs 34.1 ± 20.2, *P* < 0.001), higher right grip strength (60.8 ± 16.8 vs 49.2 ± 18.6, *P* < 0.015), and higher left grip strength (59.8 ± 18.1 vs 50.3 ± 18.0, *P* < 0.04). However, there was no difference in pinch strength.

**Conclusions:**

Our study found that ketorolac injections resulted in inferior pain reduction, functional score and grip improvement than triamcinolone injection in patients with radial styloid tenosynovitis. Future studies are required to examine the effects of ketorolac in larger group and with longer follow-up periods to further elucidate the findings of this study.

**Trial registration:**

The study was registered at Clinicaltrials.in.th (TCTR20200909006).

## Introduction

De Quervian’s disease is tenosynovitis of the first dorsal compartment described firstly Swiss surgeon Fritz de Quervain in 1895. A large UK-based study of this disease found that it commonly affected woman more than men, particularly in the 4th and 5th decades of life, with rate of 1.3% in women and 0.5% in men [1]. This condition causes severely painful radial-side wrist pain and impaired function. Treatment is directed at reducing inflammation through immobilization with the use of physiotherapeutic modalities, systemic nonsteroidal anti-inflammatory agents (NSAIDs), and/or local corticosteroid injections. Splinting with a thumb spica brace is rarely successful for long term relief, and failure and recurrence are often high [2–4]. Corticosteroid injections have been reported to provide near complete relief with one or two injections, with symptomatic relief reported by 83% of patients with a single injection in one study [5]. However, although the corticosteroid injections have a very high success rate, there are some adverse side effects in some cases including skin hypopigmentation, tendon rupture and superficial radial nerve injury, and elevated blood glucose in diabetic patients.

In recent years, ketorolac tromethamine, an injectable non-steroidal anti-inflammatory drug (NSAID) has been gaining some popularity as either an adjunct or replacement for corticosteroid injections. Previous studies have found no detrimental side effects of intra-articular ketorolac injections on articular cartilage, ligaments or kinematic function of native knees in animal models [6, 7]. For de Quervain’s disease specifically, there has been only one study, a double-blinded randomized controlled trial which demonstrated the superiority of bethamethasone injection over ketorolac injection in the treatment of De Quervain’s tenosynovitis. However, the study population was too small to provide significance [8].

There is a lack to date of high quality, randomized controlled trials to provide data regarding the effectiveness of ketorolac compared with local corticosteroid injections for de Quervain's tenosynovitis. The present study was designed to compare the effect of these drugs on de Quervain’s disease, by comparing pain scores, functional responses between and grip and pinch strength between local injections of a corticosteroid and ketorolac. Our hypothesis was that ketorolac would be superior to the corticosteroid due to the direct anti-inflammation mechanism of action and its recent history of showing better outcomes in many aspects of other orthopedics diseases.

## Patients and methods

### Study design

This was a prospective, randomized, controlled, interventional (ketorolac and triamcinolone injections), double-blinded trial performed at one tertiary center in Songkla, Thailand. The Office of the Human Research Ethics Committee, Faculty of Medicine, Prince of Songkla University approved the study protocol (IRB number EC 63–130-11–1), and the procedures in this study were performed under the Declaration of Helsinki’s ethical principles for medical research involving human participants. Written informed consent was obtained from all individual participants included in the study. The study was registered on 09/09/2020 at Clinicaltrials.in.th (TCTR20200909006).

### Recruitment

Adult patients aged ≥ 18 with radial styloid tenosynovitis were enrolled, based on a combination of the patient’s history (pain at the radial styloid), and a physical examination in which they were required to have (radial styloid tenderness, defined as pain at the radial wrist during extension or abduction of the thumb, and a positive Finkelstein’s or wrist hyperextension abduction test (WHAT test). The exclusion criteria were allergy to NSAIDs or drugs in the same group as ketorolac (e.g. diclofenac), a history of severe complications from corticosteroid or ketorolac injections including anaphylaxis, prior treatment in the last six months with a steroid injection, surgery at the same anatomical location, possible traumatic or neoplastic origin of symptoms, inflammatory joint disease at the affected wrist, inability to fill in follow-up forms or absence of self-determination, or a pre-existing condition with a high risk of an adverse drug event (e.g. ketorolac related to platelet dysfunction). All patients included in the study had a confirmed diagnosis by an orthopedic hand surgeon.

### Randomization and blinding

The patients were randomly allocated to either the ketorolac injection (group I) or triamcinolone acetate injection (group II) groups. Block-of-four randomization with computer generated random numbers was used for allocating the patients into the two groups. The allocation envelopes were opened in the out-patient clinic just before each injection. All other hand-surgeon hospital staff responsible for treating the participants after the injections were blinded to allocations, as were the patients. The investigator opened a sealed opaque envelope containing the allocation code before each radial styloid injection when preparing the injection following the type of medication indicated in the envelope.

### Technique of injection

All participants received one local injection with either 1 mL triamcinolone acetonide 10 mg/ml + 0.5 mL 1% xylocaine with adrenaline (group II: control intervention) or 1 mL ketorolac 30 mg/mL + 0.5 mL 1% lidocaine with adrenaline (group I: experimental intervention). The injected medications were pre-pared by a pharmacist and the syringe was filled away from the patient and wrapped with aluminum foil to hide the color of the solution from both patient and doctor. The local injection technique was the same in all patients. The medication was injected along the line of the tendon, just proximal or distal to the styloid, at the site of maximum pain.

### Post-injection protocol

All patients were allowed to do light activities (avoid lifting weight above10 kg), and allowed wrist motion as tolerated. Oral paracetamol 500 mg was prescribed for pain control prn. Six weeks after the injection the participants were asked to return for assessment of short-term outcomes.

### Assessment of the outcomes

All the patients were evaluated by the Thai Disabilities of the Arm, Shoulder and Hand (DASH) scale, a verbal numerical rating scale (VNRS), and grip/pinch strength. The assessments were done pre-injection and at six weeks post-injection. The Thai-DASH score is composed of two components: the disability/symptom questions (30 items, scored 1–5) and the optional high-performance sport/music or work Sect. (4 items, scored 1–5). At least 27 of the 30 disability/symptoms questions must be completed for a score to be calculated. In the study, the assigned values for all completed responses were summed and averaged, producing a score out of five for each individual item. This value was then transformed to a score out of 100 by subtracting one and multiplying by 25. This transformation was done to make the score easier to compare to other measures scaled on a 0–100 scale. A higher score indicates greater disability. The pain score used a VNRS at pre-injection and 6 weeks post-injection. A higher score indicates greater pain. The grip strength and pinch strength were recorded with a baseline hydraulic hand dynamometer from 0 to 200 lbs and a baseline hydraulic pinch gauge from 0 to 50 lbs, at pre- and 6 weeks post-injection. A higher score in these tests indicates greater grip and pinch strength.

### Statistical analysis

For a non-inferiority or superiority trial with a continuous outcome, with an effect size of 80% and a margin of 10%, 35 wrist pain patients per group were needed (alpha 5%, power 80%). All participants were included in the analysis as randomized regardless of discontinuation of treatment, lost to follow-up, or treatment conversion (intention-to-treat principle). To estimate between-group differences, we calculated improvements in the VNRS scores for pain, the Thai DASH scores, and grip and pinch strengths from baseline and 6 weeks after the trial injections. These improvements were compared using paired t-test. We reported group differences in improvement from baseline scores using a generalized linear mixed effects model. Continuous data are reported as mean ± SD when normally distributed or as median (interquartile range) when the distribution was skewed. Outcome measures were analyzed with t-test and generalized linear mixed models. We considered a *p* value of 0.05 to indicate statistical significance. The R Program Version 3.4.5 (R Foundation for Statistical Computing, Austria) was used for all statistical analyses.

## Results

**Fig. 1:**
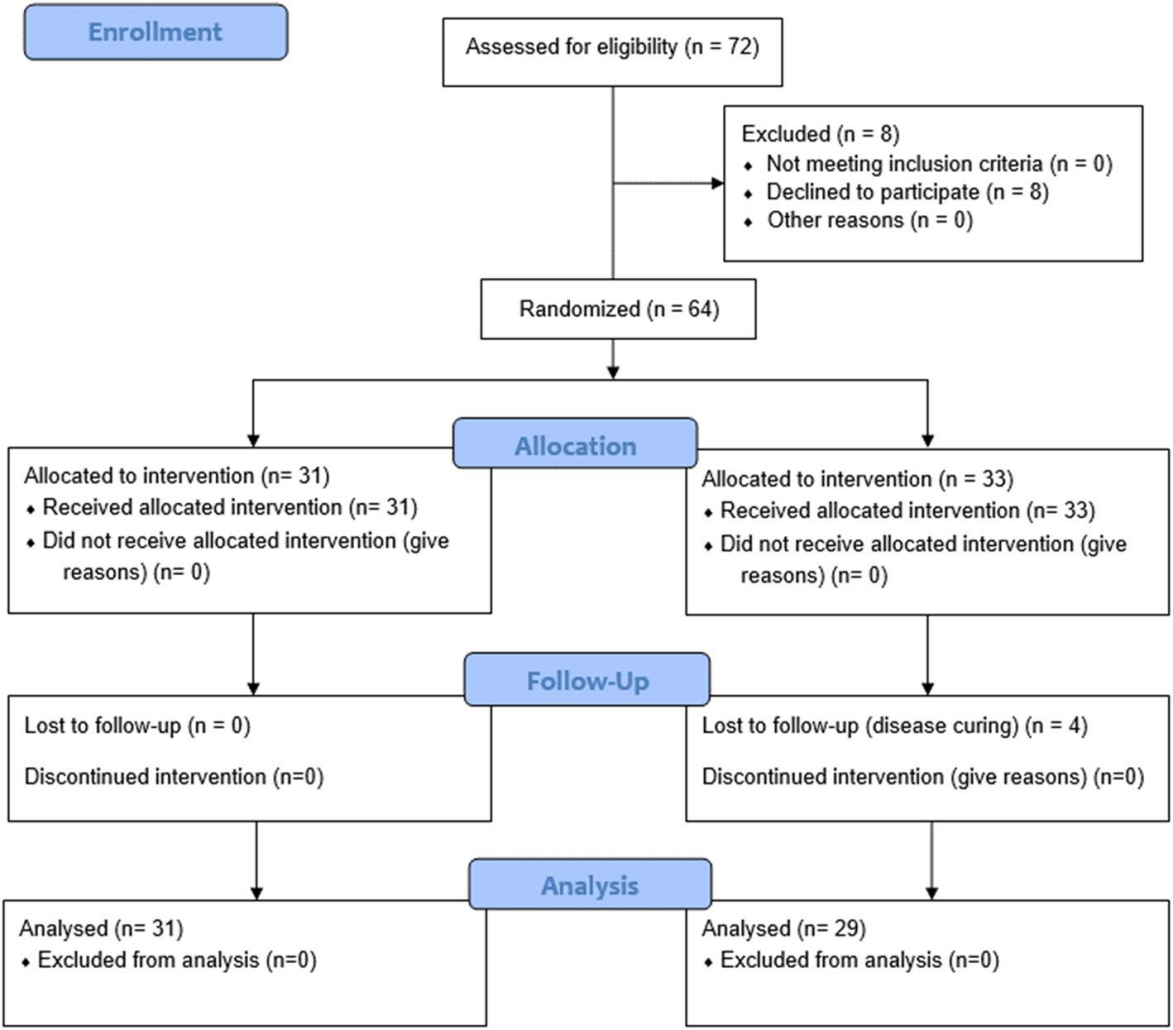
CONSORT flow diagram for the ketorolac injection versus triamcinolone injection

We screened 72 patients, eight of whom declined to participate (Fig. 1). The groups were not significantly different at baseline with regards to age, duration of symptoms, pre-operative VNRS scores for pain, DASH scores, and grip/pinch strength. At the follow-up, four participants were lost from the study because their pain had subsided (Fig. 1). Baseline data were not different between the participants who completed the study.

**Fig. 2:**
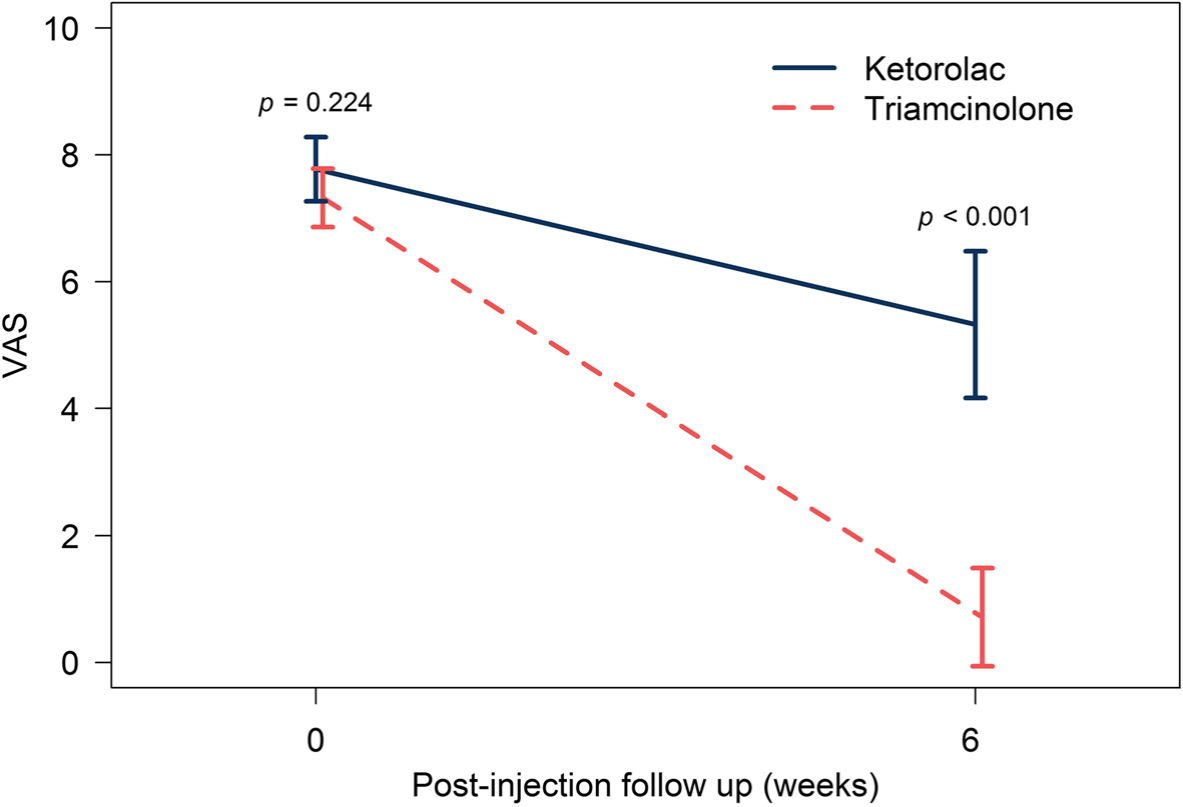
Showing pain scores at baseline and 6 weeks

A total of 64 participants were enrolled in the study and randomized according to an allocation ratio of 1:1 to the ketorolac injection treatment group (31 patients) and the triamcinolone injection treatment group (33 patients). At the follow-up visits, accounting for the 4 patients who had withdrawn, there were 31 participants in the ketorolac group and 29 participants in the triamcinolone group who completed the study and were included in the analysis (Table 1). The analysis was performed on the original assigned groups, and there was no crossover between groups. There were no significant difference in age (*p* value = 0.67) or sex (*p* value = 1), hand dominance (*p* value = 0.73), occupation (*p* value = 0.42), underlying disease(s) (*p* value = 0.42), alcohol drinker (*p* value = 1), smoker (*p* value = 0.73), or symptomatic side (*p* value = 0.95) between the groups. The average duration of symptoms for the ketorolac group and triamcinolone group were 30 ± 37 and 30 ± 47.1 days, respectively (*p* value = 0.88). There were also no significant differences in oral medications (*p* value = 0.93). All of the study outcomes including pain, DASH score, grip strength, and pinch strength are shown in Table 2. There were no significant differences in all parameters at baseline. At 6 weeks the patients in the triamcinolone group had a lower average pain score (0.7) than the ketorolac group (5.3) (Fig. 2). An additional injection at six weeks with triamcinolone was given to most patients in the ketorolac group, while no-one in the triamcinolone group (25/31 versus 0/34) needed a second injection. With the DASH test, a lower score reflects better function. Patients injected with triamcinolone had a significantly lower average score than the ketorolac group (4.4 vs 34.1). For grip strength, there were significant improvements in both groups (*p* value 0.02 and 0.04 in right grip and left grip strength, respectively). There was no difference in pinch strength between the groups at the 6-week examinations.

**Table 1:** Comparing patient baseline characteristics between the ketorolac and triamcinolone injection groups

Characteristic	Ketorolac group (*n* = 31)	Triamcinolone group (*n* = 34)	*P*-value
Age (Years)	54.5 (10)	54 (14)	0.67
Sex			1
Male	6 (17.9)	7 (18.6)	
Female	25 (82.1)	27 (81.4)	
Dominant Hand			0.73
Right	25 (80.6)	27 (79.4)	
Left	6 (19.4)	7 (20.6)	
Occupation			0.42
Self-employed	5 (16.1)	4 (11.7)	
Employee	9 (29)	9 (26.5)	
Government Officer	6 (19.4)	11 (32.4)	
Farmer	3 (9.7)	2 (5.9)	
Other	8 (25.8)	8 (23.5)	
Underlying disease			0.42
Yes	13 (41.9)	13 (38.2)	
No	18 (58.1)	21 (61.8)	
Alcohol drinker			1
Yes	5 (16.1)	6 (17.6)	
No	26 (83.9)	28 (82.4)	
Smoker			0.73
Yes	6 (19.4)	7 (20.6)	
No	25 (80.6)	27 (79.4)	
Side of pain			0.95
Right	19 (61.3)	19 (55.88)	
Left	12 (38.7)	15 (44.12)	
Duration of symptoms (days)	30 ± 37	30 ± 47.1	0.88

^*^P-value lesser than 0.05 (*P*-value < 0.05) is significant

**Table 2:** Comparing functional scores using Thai DASH score and grip/pinch strength between the ketorolac and triamcinolone groups following post-surgical decompression at 6 weeks

Outcome	Ketorolac Injection Group (*n* = 31) Mean (± SD)	Triamcinolone Injection Group (*n* = 34) Mean (± SD)	*P*-value
VNRS score at			
Baseline	7.8 (1.4)	7.3 (1.2)	0.19
6 weeks	5.3 (3.2)	0.7 (2)	< 0.001
Thai DASH score at			
Baseline	45.9 (± 17.6)	37 (± 19.8)	0.07
6 weeks	34.1 (± 20.2)	4.4 (± 6.5)	< 0.001
Right Grip strength (Pound)			
Baseline	47.9 (± 17.5)	49.9 (± 11.9)	0.61
6 weeks	49.2 (± 18.6)	60.8 (± 18.6)	0.02
Left Grip strength (Pound)			
Baseline	50.0 (± 17.7)	47.1 (± 11.8)	0.47
6 weeks	50.3 (± 18.6)	59.8 (± 18.1)	0.04
Right Pinch strength (Pound)			
Baseline	9.3 (± 4.4)	10.5 (± 3.9)	0.26
6 weeks	12 (± 8.6)	13.1 (± 4.3)	0.54
Left Pinch strength (Pound)			
Baseline	9.7 (± 3.4)	10.2 (± 3.4)	0.60
6 weeks	11.1 (± 8.5)	12.6 (± 3.3)	0.37

Values are given as mean ± standard deviation or frequency (percentage)

*VNRS* a verbal numerical rating scale, *Thai DASH* the Thailand version of Disabilities of the Arm, Shoulder and Hand

*P*-value lesser than 0.05 (*P*-value < 0.05) is significant

## Discussion

This study was undertaken to compare ketorolac and triamcinolone injections in the treatment of radial styloid tenosynovitis. Both of these drugs decrease the inflammatory process, ketorolac by inhibiting cyclooxygenase production and triamcinolone by interfering with the arachidonic acid pathway. Therefore, we hypothesized that ketorolac could have positive results similar to triamcinolone in treating radial styloid tenosynovitis. The primary outcome of this study was to compare functional scores and pain scores between the two injection techniques. Both groups reported a decrease in both the DASH scores and pain scores, meaning improvement in the overall functional scores and decreases in the pain scores.

The study found that patients who received triamcinolone had lower pain scores and better functional scores, which was consistent with a previous published study [8] that a triamcinolone injection provided better short-term outcomes in terms of reduction in pain and improving functional scores at 6 weeks. Our study found that at 6 weeks nearly 90% of the triamcinolone patients had complete resolution of their symptoms while only 40% of those who received the ketorolac reported complete resolution. A study by Chadderdon C et al. [8] also found that 52.6% and 33.3% of patients injected with ketorolac and betamethasone, respectively, required an additional injection at 6 weeks. For the DASH scores, our study found that patients injected with triamcinolone had a significantly lower average score than the ketorolac group (4.4 vs 34.1) which was consistent with another study [8] which reported that patients injected with triamcinolone had a significantly lower average score than the ketorolac group (11.1 vs 32.5). Apart from the anti-inflammatory effect, corticosteroids may affect the connective tissue and adhesions between the tendon and the surrounding peritendinous tissues by inhibiting the production of collagen, other extracellular matrix molecules, and granulation tissue in these sites [9]. These reasons support the more potent and faster action of corticosteroids compared with NSAIDs. Other studies have also compared ketorolac and a corticosteroid injection in trigger finger [10–12] and radial styloid tenosynovitis [8], and also reported the steroid injection gave better results, similar to our study, thus it may be concluded that NSAID injections may not be the best choice for use in tendon stenosis conditions. This could be because the cause of trigger finger and radial styloid tenosynovitis both result from stenosing tenosynovitis which may not respond well to anti-inflammatories and require more intensive interventions to deal with the symptoms.

However, there have been many studies which have compared the effectiveness between ketorolac and corticosteroids in various settings and conditions, including osteoarthritis of the hip [13], osteoarthritis of the knee [14], and adhesive capsulitis [15], all of which found that ketorolac provided superior results, in contrast with our results which found that triamcinolone was superior to the ketorolac. Even though our study favored triamcinolone over ketorolac in treating radial styloid tenosynovitis, there may still be a role for this drug in the treatment of radial styloid tenosynovitis patients contraindicated for steroids, for example, uncontrolled diabetes mellitus patients [10]. Another hypothesis is that ketorolac injections might reduce recurrence if given in combination with a steroid or if given the second time the patient develops recurrence symptoms, which should be investigated with further studies.

Following a ketorolac injection the patient should be monitored for adverse events such as local skin reactions, but none of our patients developed any such conditions. For the triamcinolone injection, there have been a few reports of documented adverse reactions experienced after steroid injections [8, 16–22], including flare reaction, skin hypopigmentation and sensory radial nerve impairment. In our study almost half the patients in the triamcinolone group developed some degree of hypopigmentation.

The present study is believed to be the first full published study comparing ketorolac and triamcinolone injections in treating patients diagnosed with radial styloid tenosynovitis, in terms of pain scores, functional scores, grip and pinch strength scores, and also side effects. However, several limitations must be acknowledged, including the small sample size and lack of long-term follow-up. For example, a 6-month follow-up would have been better to assess treatment response with the ketorolac group. Further studies should include longer follow-ups in order to assess the long-term effect of these drugs. The other potential limitation concerns the injection site used, at the point of maximum tenderness as indicated by the patient as this may have interfered with outcomes. In order to select a more precise injection site, ultrasonography could help identify more precisely the site of the most stenosis tenosynovitis. Further studies could be designed to assess the value of using ultrasonography-guided injections in these patients.

## Conclusions

Ketorolac injections resulted in inferior pain reduction, functional score and grip improvement than triamcinolone in patients with radial styloid tenosynovitis. Future studies are required to examine the effects of ketorolac in larger group and with longer follow-up periods to further elucidate the findings of this study.

## Data Availability

The datasets analyzed during the current study are available from the corresponding author on reasonable request.
